# Correlation of clinical findings of temporomandibular joint with serological results in rheumatoid arthritis patients

**DOI:** 10.1002/cre2.621

**Published:** 2022-06-29

**Authors:** Ranj A. Jalal, Khadija M. Ahmed, Shahla M. Saeed, Taha A. Qaradaghi

**Affiliations:** ^1^ Department of Oral Diagnosis, College of Dentistry University of Sulaimani Sulaimaniyah Iraq; ^2^ Department of Surgery University of Sulaimani Sulaimaniyah Iraq; ^3^ Bone Mineral Density and Osteoporosis Center, Ministry of Health Sulaimaniyah Internal Medicine Teaching Hospital Sulaimaniyah Iraq

**Keywords:** correlation, rheumatoid arthritis, serological test, temporomandibular joint

## Abstract

**Objectives:**

This study aimed to determine the frequency of temporomandibular joint (TMJ) involvement in patients with rheumatoid arthritis (RA) and to find out the correlation of serological tests with clinical symptoms of TMJs in RA patients.

**Patients and Methods:**

This cross‐sectional study was performed on 40 patients with RA classified into two groups according to their duration of the disease. Clinical examination as well as laboratory tests were done for participants.

**Results:**

The frequency of TMJ involvement clinically was 15% in Group A and 40% in Group B. The most frequently observed clinical symptom was facial pain (25%), and the slightest symptom was clicking (2.5%) during mouth opening. There was a positive correlation between ESR, RF, CRP and anti‐CCP and clinical sign and symptoms of TMJs in RA patients. An elevated ESR, RF CRP and anti‐CCP may indicate the presence of TMJ complains in RA patients. The chronicity of RA affects the frequency of TMJ involvement clinically, patients with longer disease duration have more clinical symptoms of TMJs. An elevated level of ESR, RF, CRP and anti‐CCP predict clinical symptoms of TMJs.

## 1 INTRODUCTION

Rheumatoid arthritis (RA) is a systemic disease characterized by chronic inflammation, joint swelling, joint tenderness, and destruction of synovial joints (Jameson, 2018). It usually affects multiple joints of the body, often starting in the peripheral joints (Jameson, 2018; Silman & Pearson, 2002).

The temporomandibular joint (TMJ) is a vital organ which closely associated with masticatory and swallowing functions, and its defect or damage severely reduces the quality of life. Generally, the TMJ pain complaints in patients with RA were recorded to be higher than 50%, the most frequent being bilateral involvement. However, it is rarely the first joint to be affected, thus, posing diagnostic challenges for the dentist (Cordeiro et al., 2016).

RA consider a systemic etiological factor with major influences on the development of temporomandibular disorders. The clinical manifestations of TMJ are often silent, so TMJ involvement in patients with RA has been ignored (Cordeiro et al., 2016).

The frequency of clinical TMJ involvement ranges from 5% to 86%, with bilateral involvement reported as the most frequent (Aliko et al., 2011; Sodhi et al., 2015).

Common clinical signs and symptoms of TMJ involvement are bilateral pain, swelling, stiffness during mouth opening, weakness of the masticatory muscles with decreased bite force, joint noises, and restriction of jaw movements (Moen et al., 2005); in the late phase of RA, ankylosis is more likely to occur (Aceves‐Avila et al., 2013).

The correlation between laboratory values of various inflammatory biomarkers causing rheumatic diseases and the progression of Temporomandibular disorders has been reported in literatures (Shim et al., 2020). Although the significant correlation indicators differed depending upon the methods and criterial used for evaluating the joint, C‐reactive protein (CRP) (Celiker et al., 1995), rheumatoid factor (RF) (Celiker et al., 1995; Lin et al., 2007), erythrocyte sedimentation rate (ESR) (Lin et al., 2007), and disease activity score (DAS) 28 showed the correlation with TMJ involvement (Hiz et al., 2012).

RF is an important diagnostic tool for assessment of RA. It is considered one of the diagnostic criteria of RA in the European League Against Rheumatism (EULAR) system (Aletaha et al., 2010; Conigliaro et al., 2016; Hodkinson et al., 2010). Anti‐CCP is a prognostic indicator for RA with a reported 80% sensitivity and 98% specificity (Marcelletti & Nakamura, 2003).

Aim of the study was to find the frequency of TMJ involvement in RA clinically and to find out correlation between clinical signs and serological results.

## 2 PATIENTS AND METHODS

### 2.1 Patients

Forty patients with RA (38 females and 2 males) who were diagnosed by rheumatology specialist according to implement criteria described by the American College of Rheumatology/European League against Rheumatism for classification and assessing the severity of the disease (Cohen & Emery, 2010) and were on medication for their RA; were involved in this study.

Patients were assigned into 2 groups based on their chronicity of RA. The first batch (A) includes 20 diagnosed patients that had RA for 1–5 years with a mean age of 49.1 ± 9.48 years, and second batch (B) includes 20 diagnosed patients that had RA for 6–10 years with a mean age of 52.15 ± 11.37 years (Table 1).

**Table 1:** Age distribution of study participants

Group	No.	Age			
		Minimum	Maximum	Mean	SD
A	20	31	66	49.1	9.48
B	20	30	74	52.15	11.37

Abbreviation: SD, standard deviation.

### 2.2 Exclusion criteria

Patients with psoriatic arthritis, osteoarthritis, history of juvenile RA, taking medication for other systemic diseases (hypertension, diabetes mellitus, hypercholesterolemia, and cancer), edentulous patients history of treatment for temporomandibular disorders (TMD); history of craniofacial trauma and patients not willing to participate.

### 2.3 Clinical examination

Clinical examination was carried out by a specialist dentist who followed the Research Diagnostic Criteria (RDC). Symptoms of pain over the face (facial, jaw, and muscle) and joints were reported, joint sounds (clicking during jaw opening and closing) were recorded, and maximal mouth opening, right/left lateral jaw excursion, and jaw protrusion was measured (Schiffman et al., 2014).

### 2.4 Laboratory tests

Erythrocyte sedimentation rate (ESR) (Sodhi et al., 2015), creatinine reactive protein (CRP) (Lapić et al., 2020), rheumatoid factor (RF) (Takeuchi et al., 2017), and anticyclic citrullinated peptide (anti‐CCP) amounts (Puszczewicz & Iwaszkiewicz, 2011) were determined for participants using standard kits from high quality licensed companies.

### 2.5 Ethical approval

Ethical Committee of College of Medicine, University of Sulaimani, Sulaimaniyah, Iraq, had revised, confirmed, and approved this study protocol with ID number 7 on January 27, 2020. This study was registered in the German Clinical Trails Register (DRKS) belongs to the World Health Organization (WHO) clinical trial registration official with ID No. (DRKS00024167). All patients had read and signed a previously designed information consent.

### 2.6 Statistical analysis

The collected data were analyzed using IBM SPSS statistics (Statistical Package for Social Sciences), version 26.0. Qualitative data were presented as number and percentage. Quantitative data were presented as mean and standard deviation. Pearson correlation coefficient were used to determine correlations between variables. Statistically significant data was considered when probability values of less than 0.05 (*p* < 0.05) were obtained.

## 3 RESULTS

### 3.1 Clinical finding

**Figure 1:**
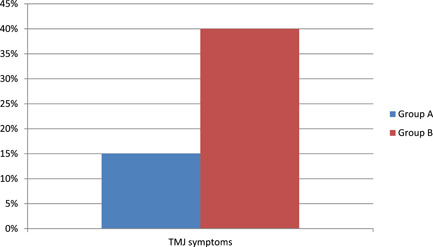
Frequency of TMJ involvement in RA patients. RA, rheumatoid arthritis; TMJ, temporomandibular joint.

Figure 1 shows that the frequency of TMJ involvement (with at least one symptom) in RA patients was 15% in Group A and 40% in Group B.

In RA patients, the jaw lock was absent in Group A while two cases (10%) of Group B had jaw lock. Three cases (15%) of Group A and seven cases (35%) of Group B complained of facial pain. Moreover, three patients (15%) in Group A felt jaw pain on the right side and two patients (10%) on the left side. Whereas five cases (25%) in Group B felt jaw pain on the right side and six cases (30%) on the left side. However, in Group B, only one case (5%) had clicking during mouth opening, and one case (5%) had pain during right lateral jaw excursion (Table 2).

**Table 2:** Frequency of symptoms of TMJs of RA patients

Group	Jaw lock	Pain face	Jaw pain		Muscle pain	Joint pain	Joint click open	Joint click close	Right lateral excursion		Left lateral excursion		Protrusion	
			RT	LT					Muscle pain	Joint pain	Muscle pain	Joint pain	Muscle pain	Joint pain
A	0	15 (3)	15 (3)	10 (2)	0	0	0	0	0	0	0	0	0	0
B	10 (2)	35 (7)	25 (5)	30 (6)	0	0	5 (1)	0	5 (1)	5 (1)	0	0	0	0
Total	5 (2)	25 (10)	20 (8)	20 (8)	0	0	2.5 (1)	0	2.5 (1)	2.5 (1)	0	0	0	0
C	0	0	0	0	0	0	0	0	0	0	0	0	0	0

Abbreviations: RA, rheumatoid arthritis; TMJ, temporomandibular joint.

### 3.2 Jaw movements

The data of mouth opening was normally distributed (*p* = 0.064), the mean of unassisted mouth opening in both groups was 39 mm with no significance difference between them (*p* = 0.289), similarly the mean of maximum unassisted mouth opening was 39 mm in both groups with no significance difference between them (*p* = 0.289) while the mean of maximum assisted opening was 39.5 mm in Group A and 39.8 mm in Group B with no significance difference between them (*p* = 0.179) (Table 3).

**Table 3:** Mean and standard deviation (SD) of mouth opening of the participants

Group	Unassisted opening				Maximum unassisted opening			Maximum assisted opening		
	Mean		SD	*p* Value	Mean	SD	*p* Value	Mean	SD	*p* Value
A	39		8.97	0.289	39	8.97	0.289	39.5	10.11	0.179
B	39		6.03		39	6.03		39.8	6.22	

Lateral jaw excursion data was normally distributed (*p* = 0.21), the mean of right lateral jaw excursion was 5.85 mm in Group A and 5.4 mm in Group B, with no significance difference (*p* = 0.719) between them. Moreover, the mean left lateral jaw excursion was 5.5 mm in Group A and 5.75 mm in Group B with no significance difference (*p* = 0.783) between them. On the other hand, the mean of mandibular protrusion was 2.05 mm in Group A and 3.2 mm in Group B with no significant difference (*p* = 0.663) between them (Table 4).

**Table 4:** Mean and standard deviation (SD) of lateral excursion and protrusion of mandible of participants

Group	Right lateral excursion			Left lateral excursion			Protrusion		
	Mean	SD	*p* Value	Mean	SD	*p* Value	Mean	SD	*p* Value
A	5.85	2.96	0.719	5.5	3.51	0.783	2.05	1.7	0.663
B	5.40	3.56		5.75	3.66		3.2	1.47	

### 3.3 Laboratory result

The ESR was positive in 15 (75%) and 17 (85%) cases of Groups A and B, respectively, without significant difference (*p* = 0.695). The RF was positive in 16 cases (80%) of Group A and 10 cases (50%) of Group B with no significant difference (*p* = 0.096). Similarly, the CRP was positive in 17 cases (85%) of Group A and 18 cases (90%) of Group B without significant difference (*p* = 1.000). The anti‐CCP was positive in 15 cases (75%) and 13 cases (65%) of Groups A and B, respectively, with no significant difference (*p* = 0.731) (Table 5).

**Table 5:** Number and frequency of participants with positive laboratory test results

Test	Group A		Group B		*p* Value
	No.	%	No.	%	
ESR	15	75	17	85	0.695
RF	16	80	10	50	0.096
CRP	17	85	18	90	1.000
Anti‐CCP	15	75	13	65	0.731

Abbreviations: CCP, anticyclic citrullinated peptide; CRP, C‐reactive protein; ESR, erythrocyte sedimentation rate; RF, rheumatoid factor.

### 3.4 Correlation of serological results with clinical findings

Table 6 show that there is a weak positive correlation between ESR and clinical symptoms while RF have weak positive correlation with joint clicking only and weak negative correlation with the remaining symptoms. CRP show weak positive correlation with all symptoms except joint clicking, and anti‐CCP show weak positive correlation with all symptoms except face pain and jaw pain only.

**Table 6:** Correlation of Serological test results with clinical symptoms of TMJs

Test	Jaw lock		Face pain		Jaw pain		Joint clicking		Pain during lateral excursion	
	* *r*	** *p* Value	*r*	*p* Value	*r*	*p* Value	*r*	*p* Value	*r*	*p* Value
ESR	0.115	0.481	0.094	0.565	0.120	0.462	0.080	0.623	0.080	0.623
RF	−0.072	0.658	−0.157	0.333	−0.107	0.512	0.118	0.470	−0.218	0.176
CRP	0.087	0.595	0.189	0.243	0.204	0.208	−0.424	0.006	0.061	0.711
Anti‐CCP	0.150	0.355	−0.082	0.616	−0.039	0.810	0.105	0.520	0.105	0.520

Abbreviations: CCP, anticyclic citrullinated peptide; CRP, C‐reactive protein; ESR, erythrocyte sedimentation rate; RF, rheumatoid factor.

* Pearson correlation coefficient.

** Significance level set at 0.05.

Table 7 show that the ESR have a weak negative correlation with unassisted and assisted opening and left lateral excursion but RF have weak negative correlation with left lateral excursion and protrusion only. CRP have weak positive correlation with all movements but anti‐CCP have weak negative correlation with all movements.

**Table 7:** Correlation of serological test results with Jaw movements

Test	Unassisted opening		Maximum unassisted opening		Maximum assisted opening		Rt lateral excursion		Lt lateral excursion		Protrusion	
	*r*	*p* Value	r	*p* Value	*r*	*p* Value	*r*	*p* Value	*r*	*p* Value	*r*	*p* Value
ESR	−0.065	0.688	−0.065	0.688	−0.098	0.549	0.100	0.546	−0.196	0.225	0.000	1.000
RF	0.073	0.655	0.073	0.655	0.026	0.872	0.132	0.422	−0.243	0.131	−0.166	0.305
CRP	0.052	0.750	0.052	0.750	0.030	0.854	0.285	0.078	0.0412	0.008	0.326	0.04
Anti‐CCP	−0.122	0.453	−0.122	0.453	−0.161	0.320	−0.214	0.191	−0.304	0.057	−0.214	0.184

Abbreviations: CCP, anticyclic citrullinated peptide; CRP, C‐reactive protein; ESR, erythrocyte sedimentation rate; RF, rheumatoid factor.

## 4 DISCUSSION

The prevalence of TMJ involvement in RA widely differs in the literature, possibly because of different examination types, criteria of patient selection, different diagnostic techniques, or involvement criteria (Savtekin & Şehirli, 2018; Silman & Pearson, 2002). this study was performed to evaluated the prevalence of TMJ involvement in RA patients and to find out correlation between serological test results and clinical symptoms of TMJs.

In present study the prevalence of TMJ involvement clinically was 15% in Group A and 40% in Group B which was lower than results reported by Sodhi et al. (2015), Savtekin & Şehirli (2018), and Akhlaghi et al. (2019).

According to previous studies, the TMJ involvement in RA follows the same destructive path as in other joints and it is correlated directly with the severity and duration of RA; therefore, the duration of RA is regarded as an aggravating factor for the involvement of TMJ (Cunha et al., 2012; Symmons et al., 1994). This finding was confirmed in the present study and the TMJ involvement was higher in patients with a longer duration of the disease (Group B).

Current study showed that 25% of patients had facial pain followed by jaw pain (20%), then clicking during mouth opening (2.5%), and muscle pain and joint pain during a right lateral excursion of the mandible (2.5%). TMJ pain was found in 65%, muscle pain in 42%, and joint sound in 51% of RA patients in a study done in Iran (Akhlaghi et al., 2019). Such differences might be related to examination methods of TMJs, variation in the number of included cases, types and frequency of drug intake in RA patients and inclusion of cases with TMD.

In the current study, the mean of unassisted opening and maximum unassisted opening of mouth were same in both groups each (39 mm) while the mean of maximum assisted mouth opening in Group A was 39.5 mm and 39.8 mm in the group **B**, which was higher than the results found by Ardic et al. (2006), who reported unassisted opening to be 37.5 mm in RA patients, however, they reported higher range of assisted opening (44.3 mm) in RA patients.

Additionally, we found that the mean of right lateral jaw excursion was 5.85 mm in Group A and 5.40 mm in Group B, while the mean of left lateral jaw excursion was 5.5 and 5.75 mm in both groups, respectively. These findings were lower than the results of a study that reported right excursion of 6.7 mm in RA, and left excursion of 6.9 mm in RA patients (Ardic et al., 2006). Most studies show a decreased range of motion in RA patients, which might be caused by reduced joint space, sclerosis, or changed condylar positioned as an adaptive procedure.

ESR is a diagnostic test commonly used to detect inflammation resulting from autoimmune diseases, although it is a nonspecific test, it is usually used to monitor the disease course (Assasi et al., 2015). The ESR level in our study was elevated in 80% which was close to Kurup et al. (2012, 2019) of Kurup et al. (2012) 87%, however, lower frequencies were detected by Yilmaz et al. (2012) and Voog et al. (2003) who found that the ESR was elevated in 28.57%, and 53% of cases, respectively.

RF is a nonspecific antibody that may be produced in some autoimmune diseases and might be present in approximately 70% of RA patients (Rindfleisch & Muller, 2005). In this study, RF was positive in 65%. This agrees with the results of Rehan et al. (2018), Gheita et al. (2012), Kurup et al. (2012), and Yilmaz et al. (2012) who found RF positivity in 64.3%, 75%, 67%, and 60.71% of cases, respectively.

In this study the CRP was positive in 35% of patients which was lower than the result found by Mortazavi et al. (2018) 46.15%.

In this study the anti‐CCP was positive in 70% of patients which was lower than the result found by Mortazavi et al. (2018) 94.23%.

There was a positive correlation between ESR and all clinical symptoms of TMJ while RF have positive correlation with joint clicking only. CRP show positive correlation with all symptoms except joint clicking, and anti‐CCP show positive correlation with all symptoms except face pain and jaw pain only. Regarding anti‐CCP and RF our outcomes agrees with results of Mortazavi et al. (2018) whose found correlation between RF and anti‐CCP with TMDs in RA patients.

## 5 CONCLUSION

The chronicity of RA affects the frequency of TMJ involvement clinically, patients with longer disease duration have more clinical symptoms of TMJs. An elevated level of ESR, RF, CRP, and anti‐CCP predict clinical symptoms of TMJs.

## AUTHOR CONTRIBUTIONS

**Ranj A. Jalal**: Conceptualization, methods, writing. **Khadija M. Ahmed**: Supervision, methods, edit writing and revision. **Shahla M. Saeed**: Supervision, edit writing and revision. **Taha A. Qaradaghi**: methods.

## CONFLICT OF INTEREST

The authors declare no conflict of interest.

## Data Availability

The data that support the findings of this study are available on request from the corresponding author. The data are not publicly available due to privacy or ethical restrictions.
